# Characterization of a variant *vlhA *gene of *Mycoplasma synoviae*, strain WVU 1853, with a highly divergent haemagglutinin region

**DOI:** 10.1186/1471-2180-10-6

**Published:** 2010-01-12

**Authors:** Awatef Béjaoui Khiari, Ibtissem Guériri, Radhia Ben Mohammed, Boutheina Ben Abdelmoumen Mardassi

**Affiliations:** 1 Laboratoire des Mycoplasmes, Département de Microbiologie Vétérinaire, Institut Pasteur de Tunis. 13, Place Pasteur B.P. 74, 1002 Tunis-Belvédère. Tunis, Tunisia

## Abstract

**Background:**

In *Mycoplasma synoviae*, type strain WVU 1853, a single member of the haemaglutinin *vlhA *gene family has been previously shown to be expressed. Variants of *vlhA *are expressed from the same unique *vlhA *promoter by recruiting pseudogene sequences via site-specific recombination events, thus generating antigenic variability. Using a bacterial stock of *M. synoviae *WVU 1853 that had been colony purified thrice and maintained in our laboratory at low passage level, we previously identified a *vlhA *gene-related partial coding sequence, referred to as MS2/28.1. The *E. coli*-expressed product of this partial coding sequence was found to be immunodominant, suggesting that it might be expressed.

**Results:**

Reverse transcription-PCR amplification (RT-PCR), using a sense primer located at the 5'-end region of the expected *vlhA *transcript and a reverse primer located at the 3' end of MS2/28.1 coding sequence, yielded a consistent amplification product showing that MS2/28.1 was indeed transcribed. Nucleotide sequence analysis of the RT-PCR product identified an 1815-nucleotide full-length open reading frame (ORF), immediately preceded by a nucleotide sequence identical to that previously reported for expressed *vlhA *genes. PCR amplifications using genomic DNA isolated from single colonies further confirmed that the full-length ORF of MS2/28.1 was located downstream of the unique *vlhA *promoter sequence. The deduced 604-amino acid (aa) sequence showed a perfect sequence identity to the previously reported *vlhA *expressed genes along the first 224 residues, then highly diverged with only 37.6% aa identity. Despite the fact that this *M. synoviae *clone expressed a highly divergent and considerably shorter C-terminal haemagglutinin product, it was found to be expressed at the surface of the bacterium and was able to haemagglutinate chicken erythrocytes. Importantly, the *E. coli*-expressed C-terminal highly divergent 60 residues of MS2/28.1 proved haemagglutination competent.

**Conclusions:**

In contrast to the previously characterized *vlhA *expressedvariants, MS2/28.1 displayed a highly divergent sequence, while still able to haemagglutinate erythrocytes. Overall, the data provide an indication as to which extent the *M. synoviae vlhA *gene could vary its antigenic repertoire.

## Background

*Mycoplasma synoviae *is an economically important pathogen of poultry, causing synovitis, chronic respiratory tract disease, and retarded growth in chickens and turkeys [1,2]. *M. synoviae *is a member of the genus *Mycoplasma *of the class *Mollicutes*, a group of wall-less Gram-positive bacteria with genomes ranging from 1358 kb to as little as 580 kb [3]. The genome sequence of *M. synoviae *strain WVU 1853 has been determined and comparative analysis with *M. gallisepticum*, another major avian pathogen, provided evidence for horizontal gene transfer between the two species, though belonging to two distinct phylogenetic groups [4,5]. Among the genes that could have arisen by horizontal gene transfer are those encoding for haemagglutinins. In avian mycoplasmas, genes encoding for these immunogenic and surface exposed proteins are the subject of considerable antigenic variability [6]. By alternating the composition of their surface proteins, mycoplasmas are thought to colonize more efficiently mucosal surfaces and become more virulent [7,8]. Haemagglutinins account among the most important surface proteins involved in colonization and virulence of avian mycoplasmas [6,9].

In *M. synoviae*, haemagglutinins are encoded by related sequences of a multigene family referred to as *vlhA *genes [10-12]. The haemagglutinins of *M. gallisepticum *(pMGA) and *M. imitans *are also encoded by multigene families related to *vlhA *[13,14]. Both organization and control of expression of *vlhA *genes are quite different between *M. gallisepticum *and *M. synoviae*. In the former species, *vlhA *genes are located in five distinct genomic regions and each gene appears to be translationally competent [14,15]. By contrast, in *M. synoviae*, all *vlhA *sequences are confined to a restricted genomic region with a unique copy being expressed in a single strain [16,17]

The uniquely expressed *vlhA *gene of *M. synoviae *yields a product that is cleaved post-translationally into a N-terminal lipoprotein (MSPB) and a C-terminal haemagglutinin protein (MSPA) [11]. Cleavage was found to occur immediately after amino acid residue 344 [17]. Both MSPA and MSPB are surface-exposed proteins and exhibit high frequency antigenic variation, but only MSPA mediates binding to erythrocytes [10,11]. Sequence analysis of several *M. synoviae *strains suggested that MSPA was more antigenically variable than MSPB [6,10,11]. Consistently, in isogenically derived *M. synoviae *clones that have lost their haemadsorbing and/or haemagglutinating activity, MSPA was no more detectable by polyclonal antisera or monoclonal antibodies, suggesting extensive antigenic variation [12].

The molecular basis underlying the generation of antigenic variants of *M. synoviae vlhA *genes has been elegantly demonstrated in a study conducted by Noormohammedi et al. 2000 [17]. It resides in the ability of a single strain to undergo high frequency site-specific recombination, owing to the availability in the genome sequence of a significant pool of pseudogenes (*vlhA*-related partial sequences). Recombination between the single complete *vlhA *gene and one of the multiple pseudogene copies ensures the creation of a new *vlhA *gene variant. To date, three expressed *vlhA *gene variants (*vlhA1*, *vlhA4*, and *vlhA5*) have been characterized in *M. synoviae *strain WVU 1853 [17]. These genes are equally sized and show extensive sequence variability in a 400-bp DNA segment in the middle of the *vlhA *sequence, suggesting that the recombination event, though introduced sequence variations, tended to preserve the overall sequence length and composition. Although it has been concluded that the potential of *vlhA *genes to vary is considerable, there is no indication as to which extent a *vlhA *gene could diverge without losing its properties.

Previous studies from our laboratory have identified in *M. synoviae *strain WVU 1853, an immunodominant *vlhA *variant (termed MS2/28.1) [18] whose haemagglutinin region displayed a dramatic sequence shift and was considerably reduced in size, relative to the previously characterized expressed *vlhA *genes (*vlhA1*, *vlhA4*, and *vlhA5*) [17]. To better evaluate the extent of antigenic variation that could be tolerated by the *M. synoviae *haemagglutinin, we sought to know whether this highly divergent *vlhA *member was properly processed and whether it remained functionally competent. Our results provide evidence that the antigenic repertoire of *M. synoviae vlhA *genes might be wider than previously perceived.

## Results

### Isolation of the MS2/28.1 fragment

The complete nucleotide sequence of MS2/28, the λ phage-derived DNA fragment (GenBank accession number MSU66315) harbouring the immuno-reactive MS2/28.1 sequence, has been previously described [18]. It is 2657 bp long and contained two partial ORFs, referred herein to as, MS2/28.1 (5' end) and MS2/28.2 (3' end) (GenBank accession numbers ORF G2149016 and ORF G2149017, respectively). MS2/28.1 lacked its N-terminal sequence, whereas the C-terminal region of MS2/28.2 was incomplete. The two partial ORFs shared 71% and 61.27% identity at the nucleotide and deduced amino acids sequences, respectively, and were separated by a 481-nucleotide non coding region (data not shown). Amino acid sequence comparisons between the two partial ORFs and the expressed *vlhA1 *gene have been previously described [11]. MS2/28.1 displayed 54.1% identity with *vlhA1*, along a 244-residue overlapping region, while MS2/28.2 showed 58.4% identity through a 495-amino acid overlapping sequence, starting at residue 260 of the *vlhA1 *gene sequence. Thus, the two partial open reading frames MS2/28.1 and MS2/28.2 are members of the *vlhA *gene family.

### Evidence that MS2/28.1 was transcribed through the unique vlhA promoter

Immunoreactivity of the λ phage MS2/28 clone was associated with the 5' end of the MS2/28.1 partial ORF. With regard to the *vlhA1 *sequence, the MS2/28.1 expressed region corresponded to the MSPA (haemagglutinin) sequence extending from residues 346 to 446, located immediately after the cleavage site.

**Figure 1:**
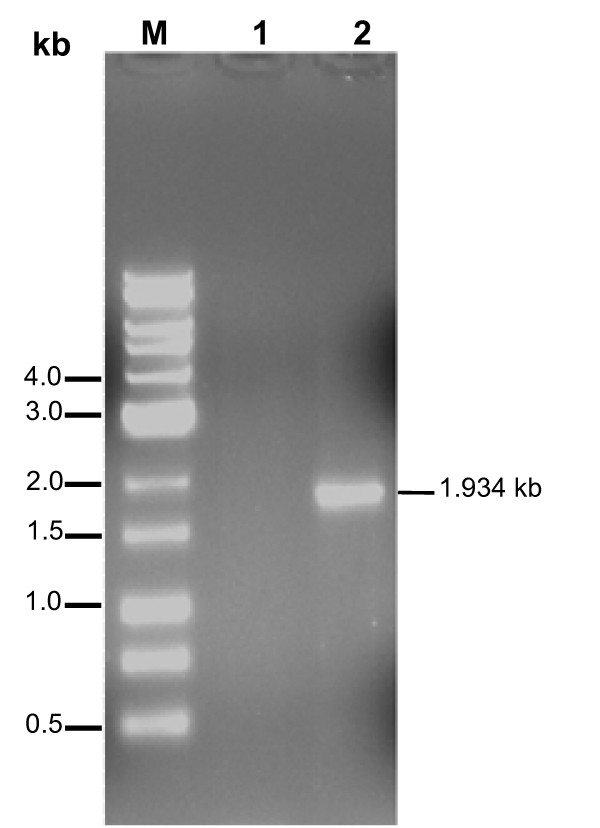
**RT-PCR targeting the unique *vlhA *derived transcript**. RT-PCR amplification of DNAse I-treated whole *M. synoviae *RNA using a sense primer (PromF) located at the 5'-end region of the expected *vlhA *transcript and a reverse primer (2/28.1Rev) located at the 3' end of MS2/28.1 coding sequence (lane 2). As negative control, PCR was directly performed on RNA without RT (lane 1). DNA size marker (1 kb) (lane M).

Given the strong immuno-reactivity of the MS2/28.1 encoded product, we hypothesised that it might be expressed in the bacterium as a *vlhA *variant. Indeed, it has been well established that in *M. synoviae*, strain WVU 1853, there exist only a single copy of the *vlhA *promoter. New variant sequences, recruited from a pool of *vlhA *pseudogenes, are placed under the control of this unique promoter via site-specific recombination [17]. Hence, we performed RT-PCR using a sense primer targeting the 5'-end of the expected *vlhA *promoter-derived transcript coupled to a reverse primer located at the 3' end of MS2/28.1 partial coding sequence. As shown in Figure 1, RT-PCR reaction yielded a DNA fragment around 1.934 kb that could not be amplified when PCR was attempted without RT reaction. This result provides evidence that MS2/28.1 was transcribed as a *vlhA *variant.

### Analysis of MS2/28.1 cDNA sequence

**Figure 2:**
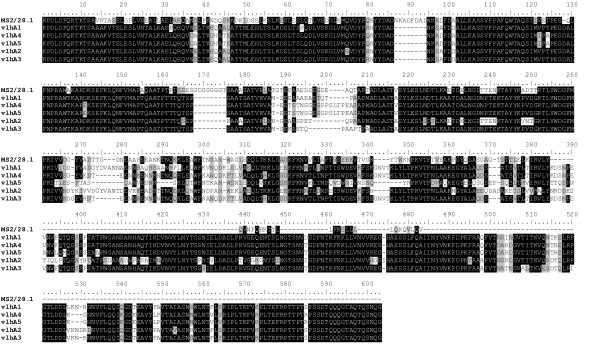
**Comparison of the amino acid sequence predicted from *M. synoviae *MS2/28.1 gene with *vlhAs *1 to 5**. Alignment of the completed full-length MS2/28.1 deduced amino acid sequence with *vlhAs *1 to 5 (GenBank accession numbers AF035624, AF085697, AF085698, AF181033, and AF181034, respectively). Identical aa regions are shaded in black while similar aa residues are shaded in grey.

To further confirm the authenticity of the RT-PCR product and to complete the full-length coding sequence of the MS2/28.1, we subjected the RT-PCR product to nucleotide sequence analysis. As expected, the 5'-end region preceding the ATG initiation codon was identical to that reported for the previously reported *vlhA *expressed genes [17]. The MS2/28.1 full-length ORF consisted of 1815 nucleotides (GenBank accession number FJ890931). The deduced 604-amino acid sequence is predicted to encode a protein with an expected molecular mass of 64.3 kDa. Sequence alignments with *vlhA1 *(GenBank accession no. AF035624), as well as with the two full-length pseudogenes *vlhA2 *and *vlhA3 *(GenBank accession nos. AF085697 and AF085698, respectively) [17] (Figure 2), showed a perfect identity along the 670 5'-end first nucleotides, corresponding to the N-terminal first 223 amino acids. Downstream from this region, a very high divergence was observed with 37,6%, 37,8%, and 38,7% aa identity, respectively. Likewise, in this region, MS2/28.1 shared only 39,8% and 38,8% identity, respectively, with the two *vlhA1 *expressed variants, *vlhA4 *and *vlhA5*, previously identified in *M. synoviae *strain WVU 1853 (Figure 2). Overall, the haemagglutinin region of MS2/28.1 was found to be considerably reduced in size (148 aa less than in *vlhA1*) and displayed high level of sequence divergence in comparison to the previously reported *vlhA *expressed genes, namely *vlhA1*, *vlhA4 *(GenBank accession no. AF181033), and *vlhA5 *(GenBank accession no. AF181034) [17].

### Demonstration that MS2/28.1 sequence is preceded by the vlhA1 promoter

**Figure 3:**
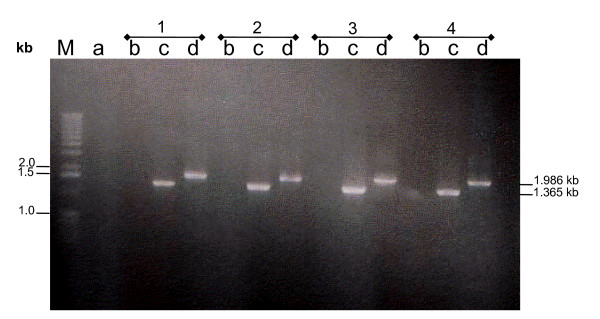
**Confirmation that MS2/28.1 is preceded by the unique *vlhA1 *promoter sequence**. Primer EXpro, which anneals to the *vlhA1 *promoter, was combined with either *vlhA1R *(lanes b) or with ORF5.1R (lanes c). No amplification from genomic DNA extracted from the four colonies was obtained with the *vlhA1*-specific reverse primer (lanes b). Expected amplicon was obtained with primers EXpro/ORF5.1R (lanes c). PCR amplification of the full length MS2/28.1 was obtained with the primers pair EXproint and 2/28.1Rev (lanes d). As negative control, PCR was performed with no genomic *M. synoviae *DNA (lane a). Lane M; DNA size marker (1 kb).

To confirm that in our bacterial stock MS2/28.1 was located downstream of the unique *vlhA *promoter sequence, we performed PCR amplifications on single colonies using oligonucleotide primers placed in the *vlhA *promoter sequence with either *vlhA1*- or MS2/28.1-specific reverse primers. As shown in Figure 3, amplicons migrating at the expected mobility were obtained solely with MS2/28.1-specific reverse primers. Sequence analysis further confirmed that the upstream sequence is identical to that of the *vlhA1 *promoter, a result consistent with the finding that MS2/28.1 is transcriptionally active and that, in its transcript, the region preceding its ATG initiation codon was identical to that reported for *vlhA1*.

### MS2/28.1 encoded full-length product is post-translationally cleaved with its C-terminal portion exposed at the bacterium's surface

**Figure 4:**
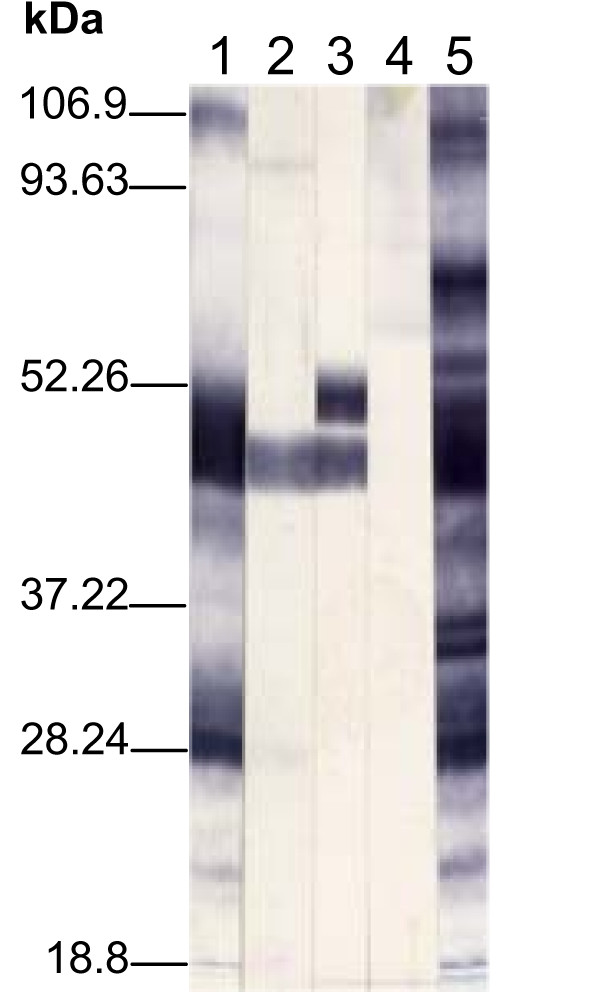
**Immunoblot of *M. synoviae *total antigens probed with antisera raised against regions A to D**. Lanes 1 to 4 show immunostaining of *M. synoviae *whole-cell proteins with antisera raised against regions A to D respectively. Lane 5 shows the reactivity of the anti-*M. synoviae *polyclonal serum. Prestained broad range protein molecular mass markers are indicated in the left margin.

**Figure 5:**
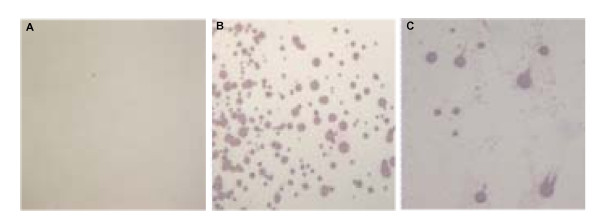
**Colony blot of *M. synoviae *probed with MS2/28.1 C-terminal region antiserum**. Immunostaining of *M. synoviae *colonies with a rabbit polyclonal antiserum raised against the MS2/28.1 C-terminal region (panel C). As negative and positive controls, the colony blots were either reacted with a preinoculation serum (panel A), or a rabbit polyclonal antiserum against whole *M. synoviae *WVU 1853 antigen (panel B), respectively.

To characterize MS2/28.1 encoded product and to examine whether it was processed similarly as the *vlhA1 *product, we generated antisera towards four bacterially expressed distinct regions of the coding sequence. The reactivity of these antisera is shown in Figure 4. The antiserum raised against the N-terminal 323 residues (region A), which represent the *vlhA1 *MSPB protein, recognized three main protein bands (Figure 4, lane 1): a diffuse 25 to 30 kDa band similar to the previously described MSPC3 [10], a second diffuse band migrating at the range of 45 to 49 kDa, typical to the previously described MSPB2 and MSPB3 proteins, and a third less intense, high molecular mass protein of 97 kDa, consistent with the uncleaved *vlhA1 *gene product. All these protein bands were revealed by the rabbit polyclonal anti-*M. synoviae *serum (Figure 4, lane 5. The monospecific antiserum raised against the 19-amino acid peptide (region B) located immediately upstream of the putative cleavage site reacted essentially with a non diffuse single band of 45 kDa (Figure 4, lane 2), identical to the *vlhA1 *MSPB protein. Thus, MS2/28.1 product was properly cleaved. This was expected because, although MS2/28.1 diverged significantly from *vlhA1*, the sequence environment of the putative cleavage site was conserved along a 17-amino acid stretch (residues 339 to 355, relative to the *vlhA1 *sequence). The monospecific antiserum to the highly reactive domain, located immediately downstream to the cleavage site (region C), reacted with only a doublet of 45 and 50 kDa (Figure 4, lane 3), similar to the two different sized bands previously described as size variants of the *vlhA1 *MSPA protein [10]. Finally, the antiserum directed against the C-terminal portion of MS2/28.1 (region D) failed to recognize a distinguishable protein band (Figure 4, lane 4). By contrast, this antiserum strongly reacted in filter colony immunoblotting assay (Figure 5C), suggesting that this C-terminal region of MS2/28.1 protein is exposed at the cell surface.

### The C-terminal highly divergent region of MS2/28.1 encoded product was haemagglutination competent

*Mycoplasma synoviae *strain WVU 1853 antigen prepared from a single colony culture with an equivalent titer of 3 × 10^7 ^CFU/ml showed haemagglutination of chicken red blood cells at a high dilution of 1:256, corresponding to a titer of 2 × 10^4 ^CFU/ml. In addition, uniform hemadsorption of chicken erythrocytes to MS2/28.1 expressing colonies was demonstrated (additional file 1). Strikingly, the *E. coli*-expressed C-terminal 60 residues of MS2/28.1 showed an haemagglutination activity. Consistently, the antiserum raised against this C-terminal highly diverged region inhibited (at a 1/00 dilution) chicken erythrocytes haemagglutination. Collectively, these data demonstrate that the haemagglutinating activity of the *vlhA *variant MS2/28.1 maps to its surface-exposed and highly divergent C-terminal 60 residues.

## Discussion

The molecular basis underlying the antigenic variability of *M. synoviae vlhA *protein, the abundant immunodominant surface haemagglutinin, has been attributed to site-specific recombination, where recruited *vlhA *pseudogene copies fuse with the unique expressed *vlhA *gene sequence [17]. Such a gene replacement mechanism, also known as gene conversion, allows a single strain of *M. synoviae *to generate a large number of variants by recruiting new sequences from a large pseudogene reservoir. This pseudogene reservoir was found to be confined to a restricted region of the genome [4,16], providing an optimal environment for site-specific recombination. The finding that MS2/28.1 gene sequence occurs in tandem with another *vlhA *related gene (MS2/28.2), suggests that it is part of this pseudogene reservoir.

Overall, the data point to the selection and clonal expansion of a WVU 1853 bacterial cell expressing a variant *vlhA *gene with an exceptionally highly divergent haemagglutinin region, comparatively to the expressed *vlhA *variant sequences described to date [17]. Indeed, all tested colonies contained an MS2/28.1 sequence located immediately downstream of the unique *vlhA1 *promoter. Comparative sequence analyses with the previously full-length *vlhA *genes, suggest that gene replacement could have occurred from aa residue 224 to the carboxy terminus. This finding adds a new 5' recombination site to the previously identified three sites (codon for residues 136, 356, and 442) [17], thus increasing the potential to generate antigenic variability.

Selection of clones expressing other *vlhA1*-related genes from a culture of *M. synoviae *WVU 1853, led to the identification of two variant clones, referred to as *vlhA4 *and *vlhA5 *[17]. These expressed variants showed a predicted protein length close to that of *vlhA1 *and diverged in their amino acid sequence by only 15% and 25%, respectively, from residue 211 to the carboxy terminus. This limited sequence variability most likely allows maintaining proper *vlhA *processing, subcellular location, and haemagglutination activity, while providing sufficient antigenic variability. By contrast, the coding sequence of the full-length MS2/28.1 ORF is considerably shorter than *vlhA1*, from which it diverged by 64%. The results showed that this highly variant sequence was properly processed, with its C-terminal highly divergent region exposed at the cell surface. In addition, the *M. synoviae *clone expressing MS2/28.1 efficiently haemagglutinated chicken erythrocytes, a property that was mapped to the surface-exposed and highly divergent C-terminal 60 residues of MS2/28.1. As far as could be ascertained, this is the first report mapping the heamagglutinating activity of a *M. synoviae vlhA *gene. The finding that the antiserum raised against this C-terminal region inhibited the haemagglutinating activity of the homologous *M. synoviae *culture, definitely confirmed that the surface exposed C-terminal 60 residues of MS2/28.1 is associated with haemagglutination. It remains to be seen whether other regions of MS2/28.1 contribute to haemagglutination.

The results described above highlight the extent to which *vlhA *genes could vary, in both the size and the sequence composition, without compromising their haemagglutination activity. Hence, comparing the expressed sequences from several naturally evolved haemagglutinin variant clones may help identifying critical residues involved in the haemagglutinating activity of *vlhA*. These variations would enable the bacterium to expose an antigenically highly divergent product to better escape the immune system [6,17]. Such a plausible consequence is presently under investigation. However, we anticipate that, during natural infection, in the face of the immune pressure, such an antigenic shift may occur frequently. It would thus be of interest to perform sequence comparisons between naturally derived *vlhA *gene sequences by focusing on their variable haemagglutinin portion. Finally, because site-specific recombination events within *vlhA *genes occur frequently through *in vitro *culture passages, inter-laboratory variations in *M. synoviae *stocks that had been colony purified are likely to exist.

## Conclusions

The present study provided an indication of the extent to which the *vlhA *haemagglutin gene of *M. synoviae *could vary without compromising the surface exposure and the haemagglutinating activity of its encoded product. We thus anticipate that the antigenic repertoire of *M. synoviae vlhA *gene could be much wider than previously thought.

## Methods

### Bacterial strains, plasmids and culture conditions

*Mycoplasma synoviae *strain WVU 1853 was obtained from the American Type Cell Culture collection (ATCC 25204 ) and grown in Frey's medium [19] supplemented with 15% (v/v) foetal calf serum. The strain was initially passaged in vitro at least 7 times before being subjected to three colony purification steps. A single colony was selected and grown. All mycoplasma cultures were then prepared from this primary stock and never exceeded two additional passages. Culture conditions and antigen preparation were performed as described elsewhere [20,21]. The mycoplasma antigens were stored at -20°C until they were needed either for Western blot, RNA or DNA extraction protocols. The growth of *E. coli *strains was carried out in LB or 2YT broths [22]. Ampicillin was added to the media at the concentration of 100 μg/ml and IsopropylB-D-thiogalacto-pyranoside (IPTG) was included for induction of protein expression at the concentrations recommended by the manufacturer (GE Haelthcare) when the screening was performed in pGEX-4T-1 plasmid transformed into *E. coli *BL21. Growth temperature were 37°C, except where indicated and growth rates were estimated by measuring the increase in OD_600_.

### Origin of the immunoreactive MS2/28 DNA fragment

Isolation and characterization of the *M. synoviae *DNA fragment MS2/28 [GenBank: MSU66315] was previously described [18]. MS2/28 contains two partial ORFs, referred to as MS2/28.1 (5' end) and MS2/28.2 (3' end).

### Reverse transcription and polymerase chain reaction (RT-PCR)

The total RNA of *M. synoviae *strain WVU 1853 was isolated from a 24-h culture, using a protocol recommended for Gram-positive bacteria [23]. Genomic *M. synoviae *DNA was eliminated from the RNA preparation using DNAse I (2,5 mg/ml) digestion for a 1-h period at 37°C. DNAse I-treated total RNA of *M. synoviae *was prepared as described above. Reverse transcription was performed at 55°C in a 20 μl reaction mixture containing 2 μg of total RNA, 4 μl of dNTP at 20 mM each, 12.5 μM of the reverse primer 2/28.1Rev (5'-GGGCGGCCGCCTACACTTGCAGTACTTGGCG-3'), 20 units of AMV reverse transcriptase and 2 μl of 10 × buffer reaction (50 mM Tris-Cl, 8 mM MgCl_2_, 30 mM KCl, 1 mM dithiotreitol, pH = 8). The first strand cDNA synthesis was allowed to proceed for 1 h followed by inactivation at 65°C during 10 min. PCR amplification was next performed using 2/28.1Rev coupled to the PromF primer (5'-GTCGACGAAATTAAGTAAATTATTAAAG-3') which anneals to the 5' end region (-120 to -98) of the expected *vlhA1*-derived transcript. The amplification reaction consisted of 30 cycles of 94°C for 120 s, 55°C for 120 s and 72°C for 120 s, followed by an extension of 72°C for 7 min.

### Cloning and sequencing of the RT-PCR product

The 1.934 kb RT-PCR product was purified and ligated into *Not*I/*Sal*I-digested pBluescript II KS+ plasmid. The ligation product was used to transform *E. coli *HB101 cells and recombinant clones were screened using restriction analysis. Determination of the nucleotide sequence was performed with the Prism Ready Reaction Dye Deoxy Terminator Cycle sequencing Kit on an ABI PRISM 377 DNA sequencer (Applied Biosystems). The cloned amplicon was sequenced in both orientations from two different plasmid clones using sequence-specific internal and plasmid-anchored primers. The sequence data were edited and aligned using the software programs BioEdit [24] and ClustalW [25].

### Confirmation of the position of the completed MS2/28.1 gene sequence relative to the unique vlhA1 promoter

Using genomic DNA extracted from single colonies as template, PCR amplifications were performed, combining EXpro (5'-CAAATTTAGTTAATTCACTTA-3'), a sense primer placed in the *vlhA1 *promoter region (-213 to -193), with either *vlhA1 *R (5'-TATTGTTTTCGGCATTATTTGCTACGTC-3'), a *vlhA1*-specific reverse primer, or ORF5.1R (5'-GCCTCCACTTCCATCTCCGCTTTCACT-3'), the MS2/28.1-specific reverse primer. To ensure that the full-length MS2/28.1 gene sequence was placed downstream of the *vlhA1 *promoter sequence, a second sense primer, EXproint (5'-ATTAACTACGTTAATTTCTTGC-3') located in the *vlhA1 *promoter region (-172 to -151) was combined with 2/28.1Rev reverse primer (5'-GGGCGGCCGCCTACACTTGCAGTACTTGGCG-3'), which anneals to the 3'-end sequence of MS2/28.1.

### Escherichia coli expression of distinct regions of the MS2/28.1 and purification of their products

The coding sequences corresponding to amino acids 1 to 324 (the N-terminal region, region A), 326-344 (region B, 19-amino acid stretch lying immediately upstream of the putative cleavage site), 354-460 (region C, the region immediately downstream of the cleavage site), and 546-604 (the C-terminal 60 residues, region D) of the full-length MS2/28.1-associated ORF (referred to as MS2/28.1) were amplified by PCR using the three primer pairs 2/28.1For (5'-GGGATCCATGAAAAATAAAAAAATTAAATT-3')-TGA1R (5'-GCGGCCGCTTGAGCTGTTCATTGGAAT-3'), TGA1F(5'-GGATCCATTCCAATGAACAGCTCAA-3')-TGA2R (5'-GCGGCCGCAGCTTTGGCTCAAGCTCTA-3'), and TGA6F (5'-GGATTCATATACTTGAAAAAATCCA-3')-2/28.1Rev (5'-GCGGCCGCCTACACTTGCAGTACTTGGCG-3') and cloned into *Bam*HI/*Not*I-restricted pGEX-4T-1 expression vector, after being verified by nucleotide sequencing. The coding sequence of the region immediately downstream of the cleavage site (354-460, region C) was obtained from a plasmid containing the MS2/28.1 segment and subcloned in the *EcoR*I site of pGEX-4T-1. The recombinant plasmids encoding regions A, B, C, and D of MS2/28.1 were electroporated into competent *E. coli *strain BL21, to produce the GST-MS2/28.1A, GST-MS2/28.1B, GST-MS2/28.1C, and GST-MS2/28.1D fusion proteins, respectively. Briefly, overnight cultures of transformed bacteria were diluted 1:100 of 2YT medium containing ampicillin (100 μg/ml) and grown at 37°C with shaking (250 rpm) to an A_600 _of 0.6. Protein expression was induced by the addition of 0.1 mM IPTG, and maintained for 4-h incubation at 30°C with vigourous agitation (250 rpm). The cells were then pelleted by centrifugation at 3000 rpm and resuspended in 1× PBS. The *E. coli *pellet was disrupted by sonication and solubilized with 1% Triton X-100 (Sigma) of 30 min. Both fusion proteins proved to be soluble and were readily purified by affinity chromotography on Glutathione Sepharose 4B Beads (GE Haelthcare), using the Bulk GST Purification Module, following the instructions of the manufacturer. The purified recombinant proteins were analysed by electrophoresis on sodium dodecyl sulfate (SDS)-12% polyacrylamide gels and allowed to react, in western immunoblotting, with a rabbit polyclonal anti-*M. synoviae *serum.

### Production of monospecific antisera to MS2/28.1 regions A, B, C, and D

Polyclonal monospecific antisera to the purified fusion proteins GST-MS2/28.1A, GST-MS2/28.1B, GST-MS2/28.1C, and GST-MS2/28.1D were raised in female New Zealand White rabbits. Pre-immune serum was collected from each animal followed by intramuscular immunization on day 1 with 200 μg protein mixed with an equal volume of Freund's complete adjuvant. Subsequent immunizations with 200 μg protein in Freund's incomplete adjuvant were given at 2 week intervals. The produced antisera were then used to identify, in *M. synoviae*, the proteins encoded by MS2/28.1.

A rabbit polyclonal anti-*M. synoviae *serum was generated as described above, using 200 μg of sonicated total *M. synoviae *antigen.

The immunization of rabbits and collection of sera were performed following the protocols approved by the Center for Biologics Evaluation and Research/Food and Drug Administration Institutional Animal Care and Use Committee.

### Identification and Characterization of MS2/28.1 encoded proteins

*M. synoviae *total proteins were separated on SDS-polyacrylamide gels and electrophoretically transferred to nitrocellulose membranes (Bio-Rad). Rabbit antisera directed either against the fusion proteins or the whole *M. synoviae *antigen were then reacted with these membranes followed by incubation with a goat anti-rabbit immunoglobulin peroxidase conjugate (Sigma). The reactive protein bands were visualized using a substrate solution consisting of 0.05% 4-chloro-1-naphtol (Sigma) in PBS containing 20% (v/v) methanol.

### Filter colony blotting

Fresh *M. synoviae *colonies growing on the surface of agar plates were transferred to nitrocellulose membrane discs (Bio-Rad). Discs were dried for 5 min at room temperature, then, they were incubated in blocking buffer (1 × PBS/5% BSA (Sigma)) for an hour. The discs were washed three times for 5 min in wash buffer (1 × PBS/0.1% BSA/0.05% Tween 20 (Bio-Rad)) and then incubated for 2 h with the primary antibody (diluted in wash buffer). After three briefly washes, nitrocellulose discs were incubated for 1 h with peroxidase-conjugated secondary antibody against rabbit IgG (GE Healthcare) diluted at 1:3,000 in wash buffer. Colony blots were visualized, after washing steps, with substrate solution containing 4-chloro-naphtol (Bio-Rad) as chromogen and the reaction was stopped by washing blots in deionised water.

### Haemagglutination and haemagglutination inhibition (HI) tests

Purified *M. synoviae *colonies were grown as described previously then harvested and adjusted to an equivalent titer of 3 × 10^7 ^CFU/ml. The cells were centrifuged, washed three times in PBS, and finally resuspended in PBS to 1/50 of the original broth volume. In rows of a U-bottomed microtiter plate duplicate serial twofold dilutions of the mycoplasma cell suspension were made in 50 μl of PBS in eight subsequent rows. To each of these wells was added 25 μl of a 0.5% suspension of chicken erythrocytes in PBS. After incubation at room temperature for 2 hs, the plate was examined for haemagglutination.

For HI assay, a 1/100 dilution of MS2/28.1 C-terminal antiserum was added to the resuspended *M. synoviae *colonies and incubated for 1 h, before adding erythrocytes.

### Colony hemadsorption assay

Distinct colonies of *M. synoviae *strain WVU 1853 derived from a single clone expressing MS2/28.1 (2.5 × 10^7 ^CFU/ml), were observed on Frey's agar after incubation for 48 h at 37°C, 5% CO_2_. The colonies were covered with 15 ml of 0.5% chicken erythrocytes in PBS and incubated for 1 h at 37°C. Agar plate was then gently washed twice with PBS and examined at low magnification under a microscope for erythrocyte adherence to mycoplasma colonies.

## Authors' contributions

ABK carried out major experimental work (PCR, RT-PCR, sequencing, sequence analysis, protein expression, production of polyclonal antisera, immunoblotting, filter colony blotting, haemagglutination and hemadsorption assays). Expression of the MS2/28.1C region and production of its monospecific antiserum were performed by GI. RBM carried out the amplification of MS2/28 5'-end cDNA and the completion of MS2/28 coding sequence. BBAM conceived, designed the study, and drafted the manuscript. All authors approved the final version of the manuscript.

## Supplementary Material

Additional file 1 **Hemadsorption of chicken erythrocytes on *M. synoviae *colonies**. Adherence of chicken erythrocytes to colonies of *M. synoviae *expressing the *vlhA *variant MS2/28.1 cultured on Frey's agar. Click here for file
