# Supplementary figures and images for: Characterization of a variant vlhA gene of Mycoplasma synoviae, strain WVU 1853, with a highly divergent haemagglutinin region
Source: BMC Microbiol. 2010 Jan 12;10:6. doi: 10.1186/1471-2180-10-6 (PMC2825196; doi:10.1186/1471-2180-10-6)

## Slide 1
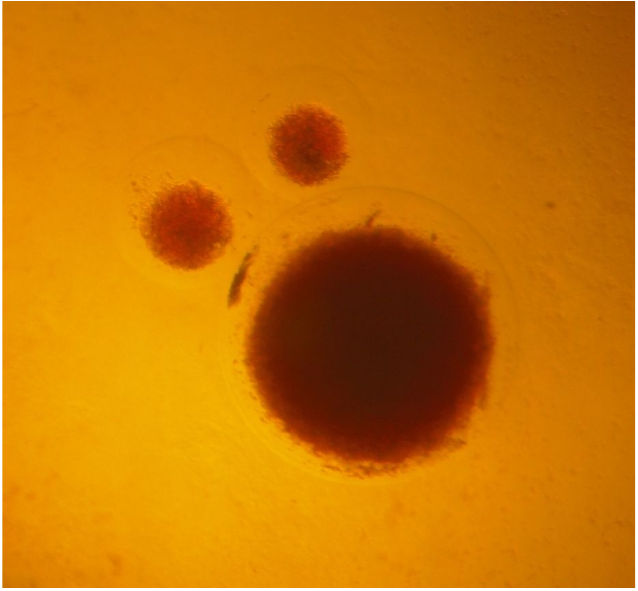

Supplement: Additional file 1 — Hemadsorption of chicken erythrocytes on M. synoviae colonies. Adherence of chicken erythrocytes to colonies of M. synoviae expressing the vlhA variant MS2/28.1 cultured on Frey's agar. [file 1471-2180-10-6-S1.PPT]
